# Fine-Tuning and the Stability of Recurrent Neural Networks

**DOI:** 10.1371/journal.pone.0022885

**Published:** 2011-09-27

**Authors:** David MacNeil, Chris Eliasmith

**Affiliations:** Centre for Theoretical Neuroscience, University of Waterloo, Waterloo, Canada; University of Sheffield, United Kingdom

## Abstract

A central criticism of standard theoretical approaches to constructing stable, recurrent model networks is that the synaptic connection weights need to be finely-tuned. This criticism is severe because proposed rules for learning these weights have been shown to have various limitations to their biological plausibility. Hence it is unlikely that such rules are used to continuously fine-tune the network *in vivo*. We describe a learning rule that is able to tune synaptic weights in a biologically plausible manner. We demonstrate and test this rule in the context of the oculomotor integrator, showing that only known neural signals are needed to tune the weights. We demonstrate that the rule appropriately accounts for a wide variety of experimental results, and is robust under several kinds of perturbation. Furthermore, we show that the rule is able to achieve stability as good as or better than that provided by the linearly optimal weights often used in recurrent models of the integrator. Finally, we discuss how this rule can be generalized to tune a wide variety of recurrent attractor networks, such as those found in head direction and path integration systems, suggesting that it may be used to tune a wide variety of stable neural systems.

## Introduction

Persistent neural activity is typically characterized as a sustained increase in neural firing, sometimes lasting up to several seconds, and usually following a brief stimulus. It has been thought to underlie a wide variety of neural computations, including the integration of velocity commands [1], [2], the reduction of noise [3], tracking head direction [4], [5], maximizing probabilities [6], and storing working memories [7], [8], [9]. The most common theoretical solution for realizing persistent activity is to introduce recurrent connections into a network model [10], [11], [12] [13]. Recently, methods have been proposed which generalize this kind of solution to any neural representation with countable degrees of freedom [14].

However, as demonstrated by [15], precise tuning of recurrent connection weights is required to achieve appropriate persistent activity in this class of simple recurrent networks. A similar observation was made earlier in numerical simulations by [16]. Specifically, in the oculomotor integrator, which has long been a central experimental target for characterizing persistent activity in a biological setting [1], [2], [17], [18], it is known that the precision of the recurrent weights required to induce drifts slow enough to match the observed behavior is quite high [19]. It has been shown that the stability of the oculomotor integrator can only be achieved by tuning the weights to within 1% of the theoretical ideal. The 1% accuracy refers to the accuracy of tuning the unity eigenvalue of the recurrent weight matrix. It can also be expressed as the ratio of the physical connection time constant, 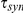, to system time constant [2]. As a result of this small 1% margin, it has been suggested that the physiological processes necessary to support such fine-tuning might not be available [20]. To achieve the observed stability, various alternative mechanisms have been explored. For instance, [21] provide evidence for a single cell mechanism that relies on cholinergic modulation. However, it is unclear if this is plausible outside of the entorhinal cortex. As well, bistability [21], [18], and multiple layers of feed-forward connections [13] have been proposed as possible mechanisms. However, the evidence supporting these more exotic possibilities in the relevant neural systems is quite weak [13].

Consequently, it is an open problem as to how real neurobiological systems produce the observed stability. The most direct answer to this question – that there are learning mechanisms for fine-tuning – has also seemed implausible. Several models that have adopted such an approach require a retinal slip signal in order to tune the integrator [22], [23], [24]. A retinal slip signal is generated by comparing the movement of the eyes to the movement of an image on the retina. If the retinal image is moving, but the eyes (and the rest of the body) are not, an error signal is generated by the oculomotor system. However, this signal is not explicitly available to the neural integrator with known connectivity, and cannot account for development of the integrator in the dark [25], [26], or the role of proprioceptive feedback [27]. Other models require an entirely non-physiological algorithm [28], or are not able to appropriately adapt to distortions in the visual feedback [29], [30]. Other accounts, that address neural stability more generally [31], [32], have not yet been shown to apply to the oculomotor integrator, and may not have the resources to do so (see Discussion).

Here we propose a learning rule that is able to account for available plasticity results, while being biologically plausible. Specifically, we demonstrate that our proposed rule: 1) fine-tunes the connection weights to values able to reproduce experimentally observed behavior; 2) explains the mis-tuning of the neural integrator under various conditions; and 3) relies only on known inputs to the system. We also suggest a generalization of this rule that may be exploited by a wide variety of neural systems to induce stability in higher-dimensional spaces, like those possibly used in the head-direction and path integration systems in the rat [33], [5], [34], [14].

## Materials and Methods

### The optimal neural integrator

To understand the results and genesis of the proposed learning rule, it is useful to begin with a standard theoretical characterization of an attractor network. The “optimal” neural integrator model used in this study is constructed using the Neural Engineering Framework (NEF) methods described in [28]. We refer to the network model as “optimal” because the NEF relies on the linear optimization to determine the connection weights (as described below). The resulting connection weights are similar to those derived by other methods [2], [15], [35], such that all such methods generate stable integrators. However, the learning rule is derived using the NEF formulation.

For simplicity, each neuron in the integrator is modeled as a spiking leaky integrate-and-fire (LIF) neuron, though little depends on this choice of neuron model [28]. The sub-threshold evolution of the LIF neuron voltage is described by
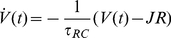
(1)where 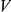 is the voltage across the membrane, 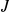 is the input current, 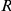 is the passive membrane resistance, and 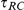 is the membrane time constant. When the membrane voltage crosses a threshold 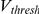, a spike is emitted, and the cell is reset to its resting state for a time period equal to the refractory time constant 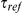. The output activity of the cell is thus represented as a train of delta functions, placed at the times of spikes 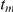 as 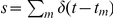. The spiking response of the cell is thus a nonlinear function of the input current 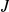, that is
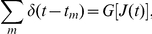
where 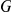 indicates the neuron model response function.

The interactions between neurons are captured by allowing spikes generated by neurons to elicit post-synaptic currents (PSCs) in the dendrites of neurons to which they project. The PSCs are modeled as exponentially decaying with a time constant of 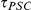:
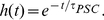
(2)For the models presented here, we assume a 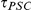 of 100 ms, which accounts for the decay of NMDA receptor PSCs, as is typical in oculomotor models [15], [20]. Notably, we have not included saturation in our model of the synapses. It has been suggested that even with long NMDA receptor time constants, there are plausible synaptic models that do not suffer significantly from saturation effects [36]. At high firing rates, small effects from saturation are evident in such models in the form of a slight roll-off of the tuning curve. This roll-off is similar to that observed when the membrane time constant of the cells is decreased. We have found our rule to provide similar results for these kinds of tuning curves (results not shown). Nevertheless, the effects of saturation, and other cellular dynamics are not captured directly by our single cell and synaptic model.

**Figure 1:**
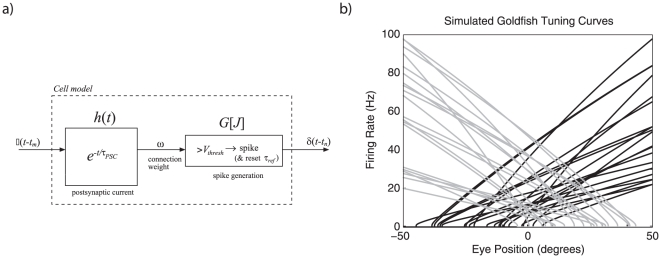
Model neurons used in the network. a) The dynamics of a model neuron coupled to a PSC model provides the complete model of a single cell. Spikes arrive, are filtered by a weighted post-synaptic current and then drive a spiking nonlinearity. b) Tuning curves for 40 simulated goldfish neurons with a cellular membrane time constant, 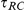, of 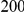 ms and a refractory period of 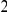 ms. Maximum firing rates were picked from an even distribution ranging from 20 to 100 Hz. Direction intercepts were picked from an even distribution between −50 and 50 degrees. The neurons were evenly split between positive and negative gains, determined by a randomly assigned encoding weight 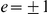.

The total current flowing into the soma of a receiving cell from the dendrites, 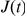, is thus determined by the input spike trains 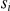 coming from connected neurons, that are filtered by the PSCs elicited by those spikes, and weighted by a connection weight between the receiving neuron and the input neurons 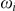:
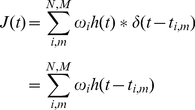
(3)where 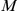 is the number of spikes from each of the 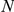 neurons connected to the receiving neuron. The somatic current then causes the receiving neuron to spike, as determined by the LIF model, and the resulting spikes are passed to connected downstream neurons. This process is depicted in Figure 1a.

To use this cellular model to perform integration it is essential to determine the appropriate recurrent connection weights 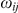. However, it is necessary to do so in light of the particular distribution of cellular responses found in the biological integrator. Here, we focus on the neurons involved in controlling horizontal eye movements, to make the problem 1-dimensional. In mammals, the horizontal oculomotor integrator is found in the nuclei prepositus hypoglossi (NPH). While it is possible to find characterizations of the cellular responses of these neurons [37], the very similar, but much simpler, oculomotor system of the goldfish is our focus of study, as it is one of the best studied oculomotor systems and has thus been more fully characterized. The cells controlling horizontal eye position in the goldfish are found in the reticular column. It is generally agreed that the goldfish integrator is a good model for the mammalian integrator despite the difference in size of the corresponding networks [15], [19].

In both mammals and fish, the relevant network of cells receives projections from earlier parts of the brain that provide a velocity command to update eye position. In addition, many of the cells in the network are connected to one another, making it naturally modeled as a recurrent network. This network turns the velocity command into an eye position command, and projects the result to the motor neurons which directly affect the relevant muscles. Thus, our model circuit consists of one population of recurrently connected neurons, which receives a velocity input signal 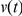 and generates a signal representing the eye position 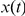.

To construct the model, we begin with an ensemble of 40 neurons (approximately the number found in the goldfish integrator), which have firing curves randomly distributed to reflect known tuning in the goldfish [15]. This neural population is taken to represent 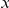, the actual position of the eye. This variable is encoded by the neural population using an encoding weight 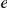, to account for directional sensitivity of the neurons. Neurons in this area have monotonically increasing firing either leftwards (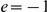) or rightwards (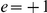). To fit the observed heterogeneity of neuron tuning in this area, we use a gain factor 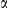. We account for the observed background firing rates of the neurons by introducing a bias current 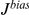. As a result of these considerations, for any neuron 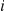 in the population, the activity produced by the neuron is given by
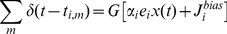
(4)where 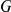 is the LIF non-linearity described by Equation 1. In essence, Equation 4 defines how eye position information is encoded into the spike patterns of the neural population.

To determine what aspects of that information are available to subsequent neurons from this activity (i.e., to determine what is represented), we need to find a decoder 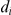. For consistency with the standard cellular model described earlier (Figure 1a), we take these decoders to be linear. This assumption, which is equivalent to having linear dendrites, is shared with most integrator models.

Optimal linear decoders can be found by minimizing the difference between the represented eye position 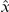 and the actual eye position 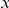 over the relevant range (see the next section):
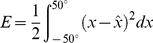
(5)where
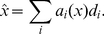
(6)The activities, 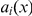 in this equation are the time-average of the filtered activity 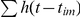 (from Equation 3) for a constant input. For the population in Figure 1b, the optimization range is 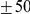 degrees and the resulting root-mean-square (RMS) error of this decoding over that range is 0.134 degrees over the 100 degrees of movement. Identifying both the encoding (Equation 4) and decoding (Equation 6) of interest provides a characterization of the time-varying representation of eye position for the population of neurons.

For the neural integrator model, it is also essential to determine how to recurrently connect the population to result in stable dynamics. [14] has shown how to determine these connection weights for arbitrary attractors. We adopt that method here, for the simple 1D case. Consider the activity of the population of neurons at a future moment in time, 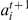. To avoid confusion, let us index that activity by 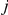; i.e., 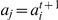. The encoding, from Equation 4, for 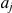 is thus
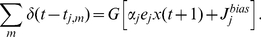
(7)At the present moment, the representation of eye position 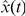, given by the decoding of the neuron activities is
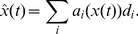
(8)Since the system should be stationary without any input, it should be the case that 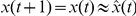 at all positions. To enforce this constraint, we substitute Equation 8 into Equation 7, giving
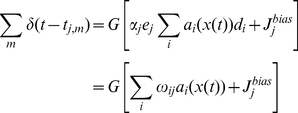
(9)where 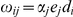. We refer to the model with these weights as the “linear optimal” model, since the weights are determined by a linear least squares optimization of Equation 5.

A network with these recurrent weights will attempt to hold the present representation of eye position as long as there is no additional input. However, even given optimal weights there are many reasons that the eye position will drift. These include representational error introduced by the nonlinearities in the encoding, fluctuations in the representation of eye position, due to the non-steady nature of filtered spike trains, and the many sources of noise attributed to neural systems [38], [39], [40]. Nevertheless, a circuit with these weights can do an excellent job as an integrator, and its performance matches well to the known properties of biological integrators [28].

Note also that this network will mathematically integrate its input. If we inject additional current into the neural population, it acts as an extra change in the eye position, and will be added to the representation of eye position. Additional input will thus be summed over time (i.e., integrated) until it stops, at which point the system will attempt to hold the new representation of eye position. In short, an input proportional to eye velocity will be integrated to drive the circuit to a new eye position. The stable representation of eye position by this circuit for different velocity inputs is discussed in the Results section.

### Derivation of optimal decoders

To complete our discussion of the optimal neural integrator, in this section we describe the methods used to compute optimal linear decoders 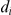 in equation 6. For generality we follow the NEF methods to determination optimal decoders under noise [28]. Specifically, we assume that the noise 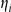 is drawn from a Gaussian, independent, identically distributed, zero mean distribution. The noise is added to the neuron activity 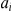 resulting in a decoding of
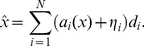
(10)To find the least squares optimal 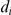, we construct and minimize the mean square error, averaging over the expected noise and 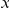:
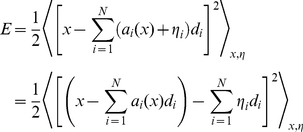
(11)where 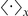 indicates integration over the range of 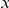. This can be thought of as multiple linear regression. Because the noise is independent on each neuron, the noise averages out except when 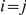. So, the average of the 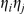 noise is equal to the variance 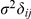 of the noise on the neurons. Thus, the error with noise becomes
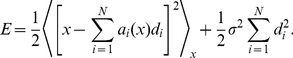
(12)Taking the derivative of the error gives
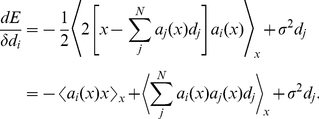
(13)Setting the derivative to zero gives
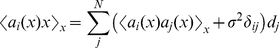
(14)or, in matrix form,
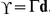
The decoding weights 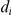 are given by
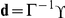
where
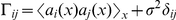


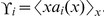
Notice that the 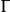 matrix is guaranteed to be non-singular, hence invertible, because of the noise term on the diagonal. In all simulations presented here the noise was taken to have a normalized variance of 0.1.

### Derivation of the learning rule

Plasticity in the neural integrator is evident across a wide variety of species, and there is strong evidence that modification of retinal slip information is able to cause the oculomotor integrator to become unstable or damped [19], [30]. Additional support for the role of tuning in the oculomotor neural integrator in humans comes from evidence of tuning within two months of birth [41], mis-tuning in subjects with developed blindness [42], and induced drift after training [43]. While evidence from experiments with dark-reared animals has shown some development of the integrator without visual feedback [25], [26], ocular stability improves when animals are provided visual feedback. Consequently, there is good evidence that some form of adaptation is active in the oculomotor integrator, and it is plausible that such adaptation would be able to support fine-tuning.

The goal of this study is to determine a biologically plausible learning rule that is able to perform integration as well as the linear optimal network described above. The learning rule derived here is based on the idea that integrators should be able to exploit the corrective input signals they receive. Empirical evidence indicates that all input at the integrator itself is in the form of velocity commands [44]. While the nucleus of the optic tract has retinal slip information, it encodes this into a velocity signal when it projects to the neural integrator [45]. Consequently, there is no explicit retinal slip signal, as assumed by past learning rules [24], [23].

**Figure 2:**
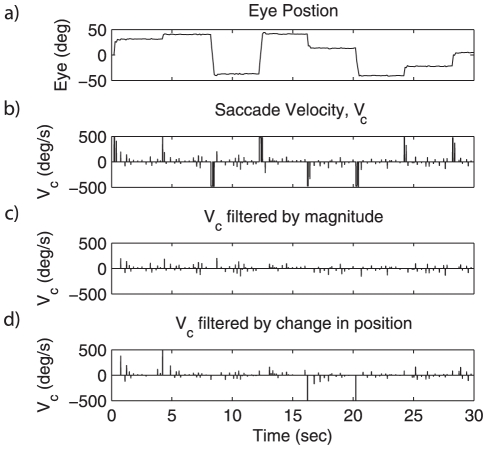
Two methods for filtering saccade commands. a) Eye position for a series of saccades. b) The saccade velocity, based on a). c) Filtering based on magnitude. This method uses Equation 15 to filter the velocity profile. This is the method adopted for all subsequent experiments. d) Filtering based on a change in position, where a change in position greater than 5 degree allows the subsequent velocity commands to pass through at a magnitude inversely proportional to the time elapsed after a movement.

In the oculomotor integrator, there is evidence of two classes of input: *intentional* and *corrective* saccades [46], [47]. [48] have argued that corrective saccades, and *not* an explicit retinal slip error, cause adaption in saccade magnitude. There are several characteristics of saccadic commands that can be used to distinguish between corrective and intentional saccades, including magnitude of velocity or change in position (see Figure 2). Because the eye is generally in the neighborhood of its target for corrective saccades, corrective saccade velocities tend to be smaller. And, since saccade magnitude is proportional to maximum saccade velocity [49], it is possible to filter saccadic velocity commands based on magnitude to identify corrective saccades. The algorithm used to filter saccade velocity 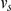 to give corrective saccades 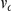 in this model is
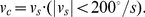
(15)That is, the corrective saccade signal consist of all velocities less than 200 degrees per second.

Furthermore [27], explains that retinal slip alone cannot account for learning in the dark and cannot incorporate proprioceptive feedback, which has some role in the long term adaption of ocular control [50]. An algorithm based on a corrective velocity signal has the potential to work with retinal slip, efferent feedback, and proprioceptive feedback, since any of these may drive a corrective eye movement. Small corrective saccades are known to occur in the dark [51].

Nevertheless, retinal slip plays an important role in the overall system. In most models of the oculomotor system, including the one we adopt below, corrective saccades are generated on the basis of retinal slip information. If the retinal image is moving, but there have been no self-generated movements (i.e., the retinal image is “slipping”), the system will generate corrective velocity commands to eliminate the slip. Consequently, the integrator itself has only indirect access to retinal slip information. Below, we show that this is sufficient to drive an appropriate learning rule.

**Figure 3:**
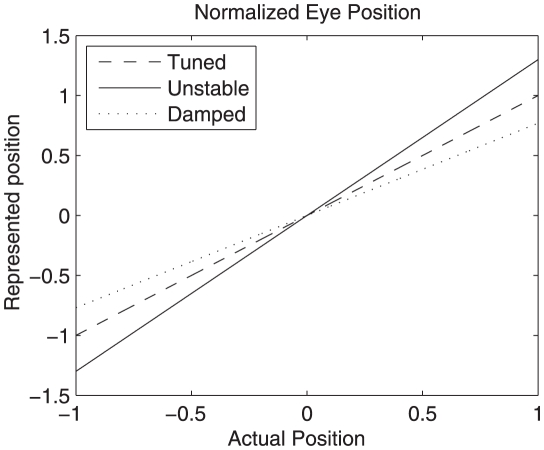
Transfer functions of actual versus represented eye position for tuned, damped and unstable networks. Eye position is normalized to lie on a range of 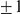. An exact integrator has a slope of 1, a damped integrator has a slope less than 1, and an unstable integrator has a slope greater than 1. Compare to Figure 9b.

Before turning to the rule itself, it is useful to first consider what is entailed by the claim that the system must be finely tuned. An integrator is able to maintain persistent activity when the sum of current from feedback connections is equal to the amount of current required to exactly represent the eye position in an open loop system. If the eye position representation determined by the feedback current and the actual eye position are plotted on normalized axes, the mapping for a perfect integrator would define a line of slope 1 though the origin (see Figure 3). This line is called the system transfer function, since it describes how the current state is transferred to future states (through feedback). A slope of 1 in the neural integrator thus indicates that the recurrent input generates exactly enough current at any given eye position to make up for the normal leak of current through the neuron membrane. In short, it means that a perfect line attractor has been achieved by the network.

However, if the magnitude of the feedback is less than what is needed, the represented eye position will drift towards zero. This is indicated by the slope of the system transfer function being less than 1. Such systems are said to be dynamically damped. Conversely, if the feedback is greater than needed, the slope of the transfer function is greater than 1 and the system output will drift away from zero. Such systems are said to be dynamically unstable (see Figure 3).

As described earlier, the representation of eye position given by equation 8 has a definite error (for the neurons depicted in Figure 1, the RMSE is 0.134 degrees). Consequently, a perfect attractor (with slope 1) will not be achievable at all eye positions. Nevertheless, it is clear from the derivation of the linear optimal integrator that changing the decoding weights 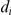 (and hence the connection weights 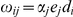) is equivalent to changing the represented value of the eye position in the network. Hence, changing these weights will allow us to more or less accurately approximate an exact integrator.

Given this background, it is possible to derive a learning rule that minimizes the difference between the neural representation of eye position 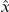 and the actual position 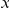. Importantly, the available corrective saccade 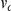 provides information about the direction in which minimization should proceed. Specifically, if 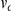 is positive the estimate must be increased so as to move towards 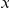; if 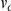 is negative the estimate must be decreased. More formally, we can express the error we would like to minimize as
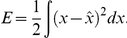
Substituting the neural representation from Equation 8 into this expression, and then minimizing it by differentiating with respect to the decoding weights 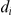 gives
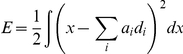


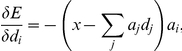
where the subscript 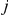 indexes the whole population and 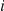 indexes the neuron currently being optimized. Note, however, that in a recurrent network 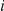 and 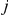 are indexing the same neurons. In addition, the connection weight dependent on 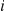 is in the postsynpatic neuron 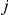. So, despite the fact that the equation is written as an optimization of 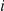, the resulting learning rule is used to tune weights in neurons 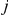 to which 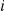 projects.

Importantly, it is now possible to substitute for the bracketed term using the negative of the corrective saccade. This substitution can be made because 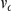 is generated by the oculomotor system so as to be proportional to, but in the opposite direction of, the difference expressed by this term (i.e., the difference between the actual and represented eye positions). Performing this substitution gives
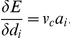
Converting this into standard delta rule form, and including the learning rate parameter 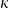, gives
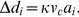
(16)This rule indicates how the decoders themselves should change in order to minimize the error.

Unfortunately, this rule is neither in terms of the connection weights of the circuit, nor local. These two concerns can be alleviated by multiplying both sides of the expression by the encoder and gain of neurons 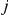, which receive projections from neuron 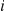


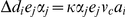


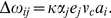
(17)The final learning rule in Equation 17 addresses both concerns. First, the NEF characterization of connection weights guarantees that the substitution of 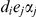 by 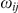 is appropriate given the definitions of the terms (as derived in Equation 9).

Second, the right-hand side of Equation 17 is in a pseudo-Hebbian form: there is a learning rate 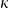, pre-synaptic activity 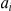, and post-synaptic activity 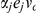. This last term is the effect of the corrective saccade on the somatic current of post-synaptic neuron 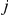, as described by Equation 7. Notably, this term is not the firing of a receiving neuron, but rather the subthreshold current that drives such firing (hence “pseudo” Hebbian). In other words, the same current used to drive the spiking activity of the neuron is used to update the connection weights. Consistent with this rule, it has been suggested in experimental work that post-synaptic potentials are not necessary for plasticity [52].

However, the current and the activity are highly correlated, as the 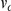 inputs must drive the neurons over threshold in order to cause the corrective saccades. Consequently, the appropriate correlations between pre- and post-synaptic firing are observed, but the postsynpatic firing does not strictly cause weight changes. As well, the rule only applies when the error term 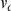 is non-zero. Hence, the corrective-saccade acts as a kind of “gate” for the connection weight changes. As a result, most accurately, the rule can be considered as a gated pseudo-Hebbian rule.

Finally, it should be noted that the integrator subject to this rule is driven by all velocity inputs as usual. Both corrective and intentional saccades determine the firing of the neurons in the integrator, and are integrated by the circuit. The mechanism that distinguishes these two kinds of saccades (figure 2), only acts to gate the learning itself, not the neural responses.

Overall, the resulting rule is biologically plausible, using only information available to neuron 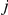. This is because neuron 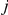: 1) receives a projection from neuron 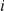; 2) is able to update the weight 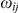; and 3) responds to input velocities, including 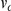, via its tuning (Equation 7). More importantly, there is no use of non-saccadic inputs (such as retinal slip). The conjunction of these properties distinguishes this rule from past proposals. We demonstrate a detailed application of this rule to the tuning of the neural integrator in the Results section.

### Generalization of the learning rule

There have been similar learning rules proposed in the literature. For example [24], propose a learning rule that uses retinal slip in place of the corrective saccades, but has essentially the same mathematical form. They also demonstrate convergence of their rule with a Lyapunov function. In an earlier cerebellar model [53], propose a learning rule in which an error provided by climbing fibers is used to tune the weight between incoming parallel fibers and Purkinje cells. This rule, too, has a similar mathematical form. So, we take the novelty of the proposed rule to lie more in its biological mapping than its mathematical form. In both previous models, there is an error signal provided on a different channel than the processed input. We have avoided this assumption, which is empirically more consistent with the circuitry of the oculomotor circuit, as described earlier.

More generally, there has been a wide variety of work examining Hebbian-like reinforcement learning (also called reward modulated Hebbian learning) that propose rules with a similar mathematical form to Equation 17 [54], [55], [56]. They are similar in the sense that the weight change is a product of an error signal, presynaptic activity and post-synaptic activity. These rules all rely on a scalar error signal that is used to drive learning. Typically this error is taken to be the reinforcement learning prediction error. But other signals are used as well. For example [24], considers the scalar retinal slip as error, and [53] assume each parallel fibre carries a single scalar value and gets an indication of the motor error. The rule we present in Equation 17 is also only applied to scalars.

However, we can extend past work by taking advantage of the NEF decomposition used in the derivation of the previous rule. In particular, the decomposition makes it clear how we can generalize the simple rule we have derived from learning scalar functions to learning arbitrary vector functions. Consider a derivation analogous to that above, which directly replaces encoding and decoding weights (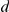 and 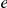) with encoding and decoding vectors (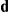 and 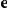), and replaces corrective saccades (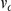) with a generalized error signal (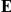). This results in a general learning rule that can be expressed as
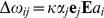
(18)where 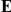 is a generalized error term (in place of 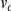, which is generated by the saccadic system).

The encoding vector 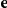 can be thought of as a generalization of the “preferred direction” vector characterized by [57]. Past work has shown how this generalization of the representation can capture many forms of neural representation throughout cortical and subcortical regions [28]. Thus, for such representations, Equation 18 suggests that the projection of an error vector onto the encoding vector can be exploited to affect weight changes of the relevant neuron. Intuitively, this suggests that the error in a vector space that can be accounted for by a given neuron, gated by its input activity, influences the relevant connection weight. This is a natural mechanism for ensuring that the neuron reduces the error that its activity affects. We demonstrate the application of this generalized learning rule to higher dimensional vector spaces after considering the oculomotor case in detail.

### The oculomotor system model

Previous learning models of the oculomotor integrator [22], [23], [24] require a retinal slip signal to drive the learning algorithm. While this signal is available to higher centers in the brain, it does not project directly to the neural integrator. Therefore, to develop a plausible learning algorithm, it is crucial to accurately model the input to each neuron. The main component of this input is the velocity signal projected to the neural integrator. Because the generation of these velocity commands is complex in itself, it is beyond the focus of the current study. As a result, we adopt the model of the oculomotor system (OMS) developed by Dell'Osso's group [58], [59], [60] to provide realistic velocity input signals. The OMS model, along with a complete description, is available for download at http://omlab.org/software/software.html.

The OMS model contains saccadic, smooth pursuit, and fixation subsystems controlled by an internal monitor. The model uses retinal signals and an efferent copy of the motor output signals to generate motor control commands. It includes the simulation of plant dynamics, and has parameters to simulate normal ocular behavior as well as several disorders. For this study, all parameters were set for normal, healthy ocular behavior.

To test our learning algorithm, we replaced the neural integrator of the OMS model with the spiking integrator model described above. To compare the tuning of our network to the experimental results of [30], it was necessary to modify the retinal feedback path of the OMS model to allow for the simulation of moving surroundings (see Results). Input to the OMS model was a target position randomly selected to lie between 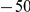 and 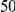 degrees. A new position was selected once every 4 s.

### Simulations

The neural integrator in this study was constructed in Simulink and embedded into the OMS model. The OMS model is available at http://omlab.org/software/software.html, and the model used in this study is available at http://compneuro.uwaterloo.ca/cnrglab/f/NIdemo.zip. A time step of 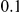 ms was used along with the first order ODE solver provided by Simulink. All simulations were run on networks of 40 neurons for 1200 s (20 minutes) of simulated time. All inputs to the model were eye position targets chosen at random from an interval of 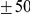 degrees, once every 4 s. At the input and output of the integrator, the signals were normalized to a range of −1 to 1 corresponding to eye position of 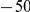 to 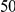 degrees. All results were collected after the 1200 s run, at which point network weights were frozen (i.e., there was no learning after 1200 s and during data collection).

The learning rule used a value of 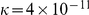 to update the weights at every time step. The value of 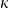 was selected by iteratively testing the model with different values of 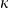 and selecting one which allowed the connection weights to converge quickly without inducing large fluctuations in the representational error. The learning rate was kept constant across all simulations.

To appropriately characterize the behavior of the model, each simulation experiment consisted of running 30 trials each with a different, randomly generated network, allowing the collection of appropriate statistics. For each trial, a new set of tuning curves for the neurons, and a new set of input functions, were randomly generated. The parameters of the tuning curves were determined based on an even distribution of *x*-intercepts over 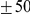 degrees, maximum firing rates picked from an even distribution ranging from 20 to 100 Hz, and a random assignment of half of the neurons to positive and negative encoding weights 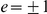. All neurons had a cellular membrane time constant, 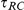, of 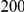 ms and a refractory period of 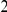 ms. All recurrent connections had a post-synaptic current time constant of 100 ms, and were modelled with a decaying exponential.

Ten different experiments were run in this manner. The first was the linear optimal integrator described above. The connections between the neurons in the linear optimal network are defined by Equation 9. All subsequent experiments start from these weights unless otherwise specified.

Several experiments add noise to the connection weights of the linear optimal integrator over time. Noise was added to the connection weight matrix 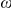 as
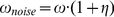
(19)at each time step for a duration of 1200 s. The noise matrix 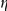 is equal to a matrix 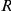 randomly selected from a normal distribution 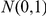 and scaled by an appropriate standard deviation 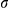 and number of time steps 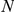: i.e., 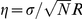. Thus, the noise is added as a standard Wiener process (i.e., Brownian motion).

In experiment 2, 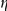 was a noise matrix with 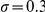, adding 30% noise over the 1200 s. Consequently, at the end of the 1200 s run using noise accumulated according to equation 19, the weights were perturbed by about 30% of their original value.

The third experiment consisted of allowing the learning rule to operate on the connection weights of the integrator networks from experiment 2. That is, after being run with the above noise and no learning for 1200 s (resulting in 30% noise), the learning rule (and no additional noise) was run for 1200 s. The fourth experiment allowed the integrator to learn while noise was continuously added to the original optimal network weights. Noise was added in the same manner as equation 19, but concurrently with learning. In this case 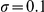 (i.e., 10% noise) was added over 1200 s. The fifth experiment allowed the integrator to learn with a combination of an initial disturbance of 30% noise (after a 1200 s run) to the optimal weights and another 5% of continuously added noise while the rule was being used. The sixth experiment examined the effects of learning starting from the linear optimal integrator, but with no noise added to the weights at all.

Experiments seven and eight were run to reproduce the results of [30]. In this study, goldfish were fixed in an aquarium where the background was controlled by a servo mechanism. The servo mechanism was programmed to rotate the background at a speed equal to eye position multiplied by a predefined gain. If the gain was in the positive direction, the network became unstable. If gain was in the negative direction, the network became damped. In our study, we directly manipulated the retinal slip feedback to simulate a moving background. Because rotation of the background in the positive direction would give the illusion of slip in the negative direction, the retinal slip in our study was modified by a gain with the opposite sign to the experimental study. We used gains of 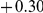 (damped) and 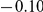 (unstable), which compare with 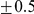 to 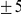 in with original study. The gains were selected to be lower than a point where they caused erratic behavior which inhibited learning (also noted by [30]). We suspect larger gains were possible in the experiments because the gain operated on an external background rather than retinal slip directly. This retinal slip signal is provided directly to the OMS model, which generates the appropriate oculomotor responses that drive the integrator.

The ninth and tenth experiments demonstrate that the rule is able to account for recovery from lesions [29]. Specifically, experiment nine shows the effect of removing a randomly chosen neuron from the network. The resulting network thus has 39 neurons. Experiment ten examines the stability of the response after applying the learning rule to the lesioned network while introducing continuous 5% noise.

### Measuring drift

Two benchmarks were used to quantify the performance of the neural integrator in these experiments. The first was root-mean-square error (RMSE) between the plot of actual feedback and the exact integrator (i.e., a line of slope 1 through the origin). This is determined by comparing the represented eye position for each possible input to the actual position given that input, and taking the difference. This provides an estimate of the representational error caused by one forward pass through the neural integrator. As a result, this error is measured in degrees. The lower this error, the slower the integrator will drift over time on average.

The second measure was the time constant, 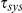, based on the average 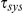 calculated from a best fit of an exponential to the response of the integrator after input pulses with a width of 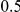 s, and heights of −2, −1, 1, and 2. This provides four evenly distributed sample drift points for each network, which are averaged to provide the final estimate.

Data was collected for 30 randomly generated networks (i.e., neuron parameters are randomly chosen as described above) and used to calculate a mean and 95% confidence interval (using bootstrapping with 10,000 samples) for both RMSE and 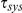. For the calculation of 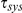, the absolute value was used to calculate the mean and confidence interval, and the sign was later found by summing 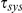 over the 30 trials.

## Results

### Application of the learning rule to the oculomotor integrator

To demonstrate the effectiveness of the proposed learning rule (equation 17), we present the results of the ten experiments in order to benchmark the system and reproduce a variety of plasticity observations in the oculomotor system.

**Figure 4:**
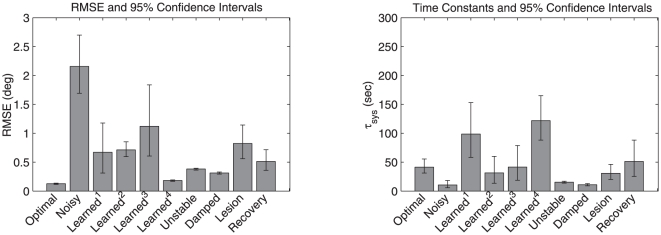
Bar graphs for the experiments described in the main text. a) RMSE and b) the magnitude of 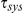 for each experiment. The error bars indicate the 95% confidence intervals as reported in Table 1.

The summary results of the ten experiments are shown in Table 1. The time course of various example networks are described subsequently. All results in the table are averaged over 30 network models with randomly chosen neuron properties (see Materials and Methods). Figure 4 reproduces these results as a bar graph, for visual comparison. In each case, the mean and 95% confidence intervals are presented.

**Table 1:** RMSE and system time constant (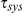) for the experiments described in the main text.

		RMSE (degrees)		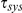 (s)	
Experiment		Mean	CI	Mean	CI
1	Optimal	0.129	0.115–0.138	(+) 41.4	31.2–55.6
2	Noisy	2.156	1.693–2.699	(+) 10.6	5.85–18.2
3	Learned+Perturb^1^	0.671	0.312–1.178	(+) 98.7	58.5–153
4	Learned+Noise^2^	0.712	0.595–0.854	(−) 31.6	13.5–60.1
5	Learned+Perturb+Noise^3^	1.120	0.606–1.838	(+) 41.4	18.9–78.8
6	Learned+NoNoise^4^	0.183	0.170–0.193	(+) 122	88.1–165
7	Unstable	0.382	0.364–0.395	(−) 15.5	13.8–17.1
8	Damped	0.313	0.294–0.329	(+) 10.9	9.19–13
9	Lesion	0.824	0.561–1.142	(+) 30.8	20.2–46.2
10	Recovery	0.513	0.359–0.716	(−) 51.3	25.4–88.1

1 After an initial disturbance (30%) to connection weights.

2 With continuous noise (10%) added to connection weights.

3 After an initial disturbance (30%) and continuous noise (5%).

4 No noise.

CI is the 95% confidence interval. Positive and negative signs indicate the direction of drift; towards and away from zero respectively.

The root-mean-squared error (RMSE), measured in degrees, quantifies the average difference between the exact integrator transfer function (a straight line) and the estimated transfer function of the model circuit (as described in Materials and Methods). Typically, higher RMSE means more rapid drifting (between stable points) since error accumulates more quickly. However, the transfer function is estimated using rate model approximations to the simulated spiking neurons, so this relationship is not guaranteed to hold. Consequently, we also report the absolute value of the system time constant, which is indicative of the speed at which the system drifts (see Materials and Methods). The sign, shown in brackets, indicates the direction of drift. A negative sign indicates a drift away from midline (zero), and a positive sign indicates a drift towards midline. All time constants are in seconds.

**Figure 5:**
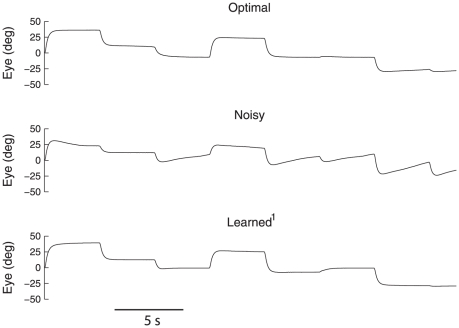
Generated eye movements of example networks. The linear Optimal, Noisy (30% perturbation to connection weights), and Learned+Perturb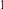 (after 1200 s of learning from the Noisy state) networks are shown for 30 s with the same saccade regime.

**Figure 6:**
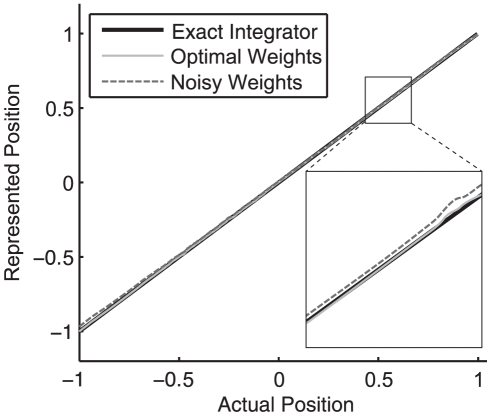
A comparison of the exact integrator, linear Optimal and Noisy transfer functions over a normalized range. The linear Optimal network is closer to the exact integrator over the range of eye positions. Although deviations of the Noisy network from the exact integrator are small, the effects on stability are highly significant (see Table 1 and Figure 5). Magnified regions are to aid visual comparison.

The four experiments in which the system learns under a variety of noise profiles demonstrate the robustness of the rule. As is evident from Table 1, the addition of 30% noise to the connection weights (Noisy) increased the RMSE by over an order of magnitude. Consequently, the mean time constant was reduced from 41.4 s to 10.6 s. The time traces of the eye position for example linear Optimal, Noisy, and Learned+Perturb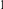 networks are shown in figure 5. As well, a comparison of the transfer functions of the exact, Optimal, and Noisy integrators is shown in Figure 6. Together, these graphs demonstrate that after the initial perturbation, the network no longer performs integration properly. However, with the introduction of the learning rule, the integrator is able to overcome the noise.

In fact, as shown in Figure 4 the tuned network can be more stable than the linear Optimal case (compare Learned+Noise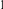 or Learned+NoNoise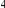 to Optimal). There is no overlap in confidence intervals, making it clear this is a strong effect. In other words, using the learning rule can tune the network better than “optimal” (see Discussion). In both cases, this improvement beyond the Optimal case occurs when there is no noise during the learning period.

Consequently we consider the rule under continuous noise. With the continuous addition of 10% noise (Learned+Noise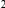), the integrator is also able to retain a similar time constant to the linear Optimal case, though there is a slight increase in the variability of the drift over the 30 test networks. This demonstrates that the system is robust to continuous noise, but does not show that it can retune after an initial disturbance and with continuous noise.

In the case of combined initial and continuous noise (Learned+Perturb+Noise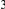), the learning rule maintains the same mean as the linear Optimal case, though again with a slight increase in variability. We found that the continuous noise in this case had to be reduced (to 5%) to allow retuning from the initial perturbation.

Taken together, these results suggest that the learning rule is as good as the optimization at generating and fine-tuning a stable neural integrator. In fact, with no noise (Learned+NoNoise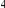), the learning rule can tune the integrator to have a much longer time constant than the linear Optimal case. This is because the model that is optimized has various assumptions about neural properties which are violated in the model (e.g., rate versus spiking neurons). In short, the learning tunes the network better than the standard optimization – we return to this point in the discussion.

**Figure 7:**
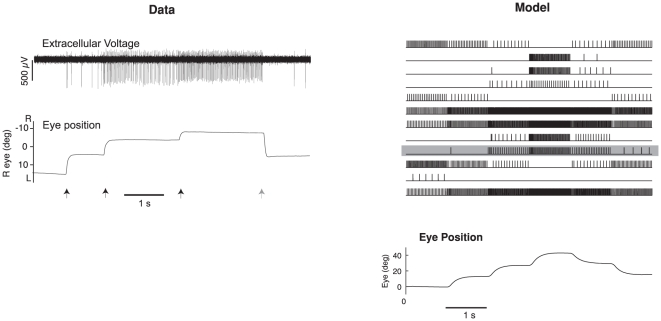
Comparison of goldfish integrator neurons from electrophysiological recordings and the simulation after tuning with the learning rule. A single raw recording is shown on the left, along with the corresponding eye trace. Arrows indicate times of saccade (black right, grey left; adapted from [30]). The right shows 14 neurons randomly selected from the model population after tuning with the learning rule. Neurons in the model have similar kinds of responses as the example neuron. One is highlighted in grey.

The results can also be compared to the goldfish integrator, which has empirically measured time constants that range between 29 s and 95 s, with a mean of 66 s [19, fig. 6]. As shown in Table 1, this compares well with experiment 5, in which the simulation has been tuned after an initial disturbance, and constant ongoing noise of 5% (mean 41.4 s, CI: 18.9–78.8). To get a better understanding of the temporal behavior of the simulations as compared to the biological system, Figure 7 shows a 6 s run with several saccades in both systems. The simulation effectively reproduces the kinds of responses seen in integrator neurons, and the related eye movements.

**Figure 8:**
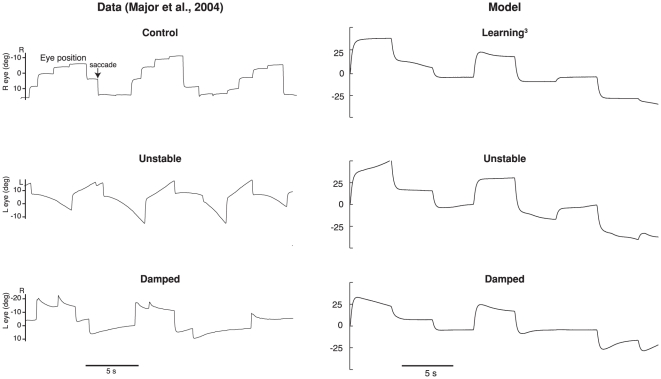
A comparison of the simulated detuning experiments with experimental data [**30**]. The top trace is for the control situation, which for the model is tuning after a 30% perturbation and 5% continuous noise. The middle trace shows the unstable integrator, and the bottom trace shows the damped integrator. The goldfish traces are from animals that had longer training times (6 h and 16.5 h respectively), than the model (20 min). Both the model and experiment demonstrate increased detuning with longer training times (not shown), and both show the expected detuning (drift away from midline for the unstable case, and drift towards midline in the damped case).

The results from the unstable and damped experiments reproduce the major trends observed in the experimental results, as shown in Table 2 and Figure 8. For the Unstable case, the learning rule demonstrates a large difference between the tuned and untuned networks, going from 41.4 s to an average value of −15.5 s (drift is away from zero, see Figure 8). The 95% confidence interval is also well outside that for the any of the linear Optimal or Learned cases. This compares well to the experimental change reported. The animals in [30] were trained between 20 min and 16.5 h, with averages only reported for animals after 1 h or more of training. Simulations of that length were not feasible, and so all simulations were run for 20 min of training. Hence, slightly smaller changes are expected. However, for both the simulations and the experimental system, longer detuning resulted in faster time constants. A similarly sized change is evident in the damped case, which shows an average reduction to a time constant of 10.9 s (drift towards zero) for the simulation and 7.7 s for the experiment (see Figure 8).

**Table 2:** A comparison of the time constants observed in our model to experimental results.

	Simulation	Empirical Data
Experiment	(20 min training)	(1 h or more training)
6 Learned+Perturb 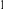 /Control	41.4	66.0 [19]
7 Unstable	15.1	4.3 [30]
8 Damped	10.9	7.7 [30]

All values are the reported 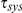 in seconds.

**Figure 9:**
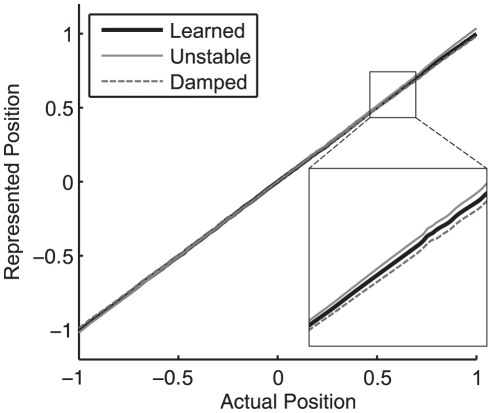
A comparison of the Learned+Perturb+Noise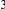, Unstable and Damped transfer functions. The slope of the Unstable network is greater than 1 and that of the Damped network is less than 1. The re-tuned networks demonstrate the expected drifting behavior (see Figure 8 and Table 1).

Figure 9 compares the transfer functions for the Unstable, Damped, and Learned+Perturb+Noise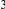 networks. Notably, a small deviation from the transfer function of the exact integrator causes reasonably rapid unstable or damped performance. The zoomed in sections of this figure make the differences between pre and post-tuning more evident. It is crucial to show the entire transfer function, however, as it demonstrates that the time constant change is smooth across all eye positions (the transfer functions are approximately straight lines). The same is observed experimentally [30].

One noticeable difference between the experiments and simulations is the variability in the system after training. While the standard deviations for the experimental results are not available, the range of one correctly tuned experiment is reported as being from −31 s to 15 s [30], which is a much greater spread than observed in the simulations. There are several possible reasons for this much wider variance. While we have attempted to match the variability of the tuning curves, there are several other parameters kept constant across simulations that are likely varying in the biological system, such as synaptic time constants, and learning rates. These are fixed in the simulations, as we do not have experimental estimates of the distributions of these parameters. Nevertheless, the important features of detuning, including the direction and extent of the detuning are reproduced in the simulations.

**Figure 10:**
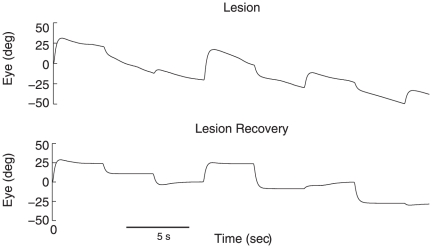
Performance of the integrator before and after lesioning the network. Severe drift is evident after randomly removing one of the 40 neurons. After 1200 s of recovery with the learning rule under 5% noise, the time constant improves back to pre-lesioning levels.

To simulate the lesion of a single neuron, the network was tuned to the linear optimal weights before a single neuron was removed. Lesioning a neuron resulted in an increase in RMSE from 0.129 to 0.824 and a decrease in time constant to about 10 s. To demonstrate the recovery process documented by [29], the learning rule was then run on the lesioned network under 5% continuous noise. The system was able to recover to a system time constant of 51.3 s on average. The temporal properties of the network are shown before and after lesioning in Figure 10.

### Application of the generalized learning rule

In other work, we have shown how this characterization of the oculomotor integrator as a line attractor network can be generalized to the family of attractor networks including ring, plane, cyclic, and chaotic attractors [14]. These attractors have been implicated in a wide variety of biological behaviors including rat head-direction control (ring), working memory and path integration (plane), swimming and other repetitive movements (cyclic), and olfaction (chaotic). The generalized learning rule described above applies in a straightforward manner to these other cases.

**Figure 11:**
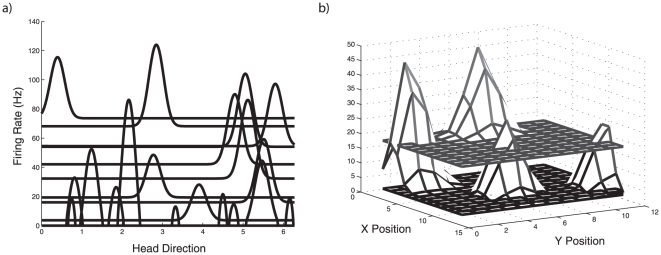
Tuning curves in two function spaces. a) Gaussian-like tuning curves of 20 example neurons in a one-dimensional function space (7-dimensional vector space). These are tunings representative of neurons in a head-direction ring attractor network. b) Multi-dimensional Gaussian-like tuning curves of four example neurons in a two-dimensional function space (14-dimensional vector space). These are tunings representative of neurons in a subicular path integration network.

For example, the ring attractor is naturally characterized as a stable function attractor (where the stabilized function is typically a “bump”), as opposed to the scalar attractor of the oculomotor system. Similarly, a 2D bump attractor, which has been used by several groups to model path integration in rat subiculum [61], [34], can also be characterized as a function attractor in a higher dimensional space. A function space can be represented as a vector space, and so we can apply the generalized learning rule to tune this network. Example tuning curves in these function spaces are showing in figure 11.

Analogous simulations to the oculomotor Learned+Perturb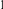 case were constructed in the Nengo neural simulation package to characterize tuning of head direction and path integrators networks (Nengo was used as it executes these simulations more quickly than Matlab. They are available at http://compneuro.uwaterloo.ca/cnrglab/f/NINengoDemos.zip). Specifically, for the head direction system, neurons were randomly assigned unit encoding vectors 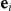 in a 7D vector space, to define encodings as in equation 8. Initial optimal weights were calculated as defined in equation 9, using encoding and decoding vectors rather than weights (i.e. 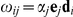). Both the represented vector space is mapped to the 1D function space using a cyclic orthonormal basis 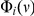: e.g., to get the encoding functions we compute 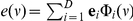, where 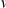 is the 1D spatial variable and 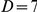. The same process is followed for the path integrator using a 14D vector space and 2D function space.

**Figure 12:**
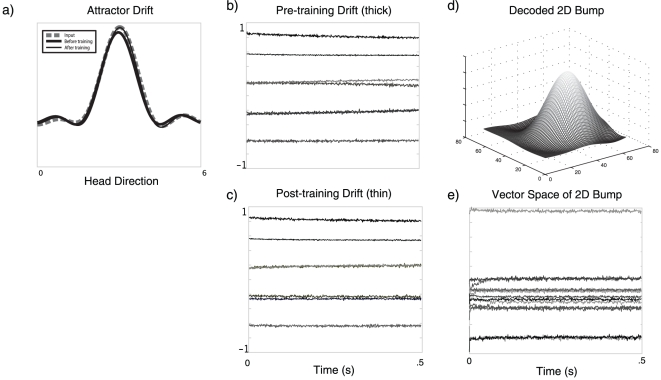
Simulations of tuning attractor networks in higher dimensional spaces. a) The input (dashed line) along with the final position of the representation after 500 ms of drift for pre-training (thick line) and post-training (thin line). b) The pre-training drift in the vector space over 500 ms at the beginning of the simulation for the bump (thick line in a). d) The drift in the vector space over 500 ms after 1200 s of training in the simulation (thin line in a). Comparing similar vector dimensions between b) and c) demonstrates a slowing of the drift. d) A 2D bump in the function space for the simulated time shown in e), after training. e) The vector drift in the 14-dimensional space over 500 ms after training.

Figure 12 shows example results from these simulations, using the generalized learning rule. The models are very similar since both can be realized by different stable structures in a vector space [14]. Hence, the simulation setups are identical, except the head direction network has 7 dimensions and 700 neurons, and the path integration network has 14 dimensions and 1400 neurons. Neurons have the same parameters as in the oculomotor simulation, except that encoding vectors are chosen to tile the appropriate spaces (analogous to choosing encoding weights of 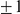 in the oculomotor network).

As shown in Figure 12, the same trend of improving the time constant over a 1200 s run is evident in the other networks. For the ring attractor, the time constant improved from 7.69e
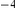
s to 2.9 s. The increase in time constant is evident in the decrease in the amount of drift in Figure 12 between the beginning and the end of the simulation. In this figure, we have also shown the difference in drift in the function space. A similar trend, with lower time constants, was evident in the head direction network over 1200 s of training (from 7.69e
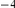
s to 1.1e
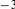
s). This small change in the time constant is not visually evident in plots like those for the ring attractor. Instead we have shown the representation of a stable 2D bump at the end of training. It is clear from these simulations that the number of neurons per dimension is not sufficient to achieve a similar level of stability as seen in the oculomotor integrator in the higher dimensional spaces. This is not surprising, as the number of neurons required to achieve a similar RMSE goes to the power of the number of dimensions of the space. This means that stable representations in higher-dimensional spaces are much more difficult to achieve for a given number of cells. Exploring the relationship between the number of neurons, the dimensionality of the space, and stability properties, and properly quantifying the behaviour of the learning rule in detail in these spaces remains future work.

These simulations are intended only as a proof-in-principle that the learning rule generalizes, and are clearly inaccurate regarding the biological details of both systems (e.g., neuron parameters should not be the same as the oculomotor integrator). More importantly, the generalized error needed in each simulation 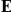 needs to be identified in each case. Our assumption that there is drift information analogous to the oculomotor integrator may or may not be biologically plausible. Consequently, in each case there remains important questions regarding the existence and source of the required error signals. These questions go well beyond the scope of the current paper. However, these simulations do demonstrate that the same kind of learning rule can be used to tune a wide variety of attractor networks in higher-dimensional spaces.

## Discussion

The simulations described in this paper demonstrate one possible solution to the problem of fine-tuning in neural integrators. The oculomotor model was able to achieve and maintain finely-tuned connection weights through a biologically plausible learning algorithm. Specifically, the learning rule allowed recovery from large perturbations of connection weights, continuous perturbation of connection weights, and the lesioning of cells. Not surprisingly, these results are in agreement with other experimental findings that suggest that feedback plays an important role in the behavior of the oculomotor integrator [42], [43], [41].

Consideration of the learning rule suggested here demonstrates that on-line fine-tuning is a viable *in vivo* mechanism for explaining the stability of the neural integrator. Specifically, this rule improves upon existing oculomotor learning models [29], [22], [23], [24] by expressing the modification of synaptic weights in terms of information that is known to be available to each neuron. Furthermore, this rule is able to explain not only robustness to random connection weight noise, but also several experimental findings related to other forms of perturbation. For instance, unlike rules that strictly enforce stability [32], this model is able to replicate the de-tuning observations described by [30]. The learning rule is able to tune the integrator in response to distorted visual feedback in a way comparable in terms of both required training time and degree of instability/damping observed [30]. As well, the system is able to tune the integrator after cell death, which has been observed empirically [29]. Consequently, this rule provides a plausible mechanism for solving the fine-tuning problem, without relying on less well-established mechanisms (e.g., [20], [13]).

### Optimality of linear methods

Using feedback to tune the integrator results in learned connection weights that produce the same or even longer time constants than the theoretically derived linear optimal connection weights, despite a significantly larger RMSE (compare experiments one, three, four, five, and ten). This is likely because the calculation of linear optimal weights does not account for dynamics of the eye or the spiking non-linearities in the neurons (see Materials and Methods).

In contrast, the learning algorithm is employed alongside the simulation of the oculomotor plant and single cell dynamics, so the learned weights are calculated for a more complete model rather than an approximation to that model. The effect of these approximations is most directly demonstrated by experiment six, in which the learning rule tunes the system with no noise. In this case, the average learned time constant is three times longer than that of the linear optimal network, even though the RMSE is higher as well. This is true regardless of how much noise is assumed during the optimization process (results not shown). This suggests that typical theoretical methods for tuning connection weights are not generally “optimal” in fully spiking network models.

### Empirical consequences

Despite the limitations of these theoretical optimization methods, they are important for allowing the network to be in a neighbourhood where it can be fine-tuned. This rule will not tune a completely random network with large amounts of continuous noise, for instance. As a result, one empirically testable consequence of this model is a characterization of the maximum amount of noise such a mechanism can tolerate. In particular, the system is robust under 10% continuous noise, or under 30% initial and 5% continuous noise. This makes it reasonable to expect that the amount of continuous noise of this type in the system would be on the order of 5–10% (over twenty minutes). While this degree of robustness is significant, it remains to be seen how robust the biological integrator is to these same kinds of perturbation, and how severe intrinsic perturbations in the system are. Given our model, we suggest that the magnitude of intrinsic perturbations could be determined by examining the extent and speed of detuning when corrective saccades are inhibited or removed. For instance, under the same 10% continuous noise for 200 minutes with no corrective saccades, the average system time constant becomes 7.68 s (confidence interval: 4.67 s–11.8 s) in the model. We leave for future consideration careful characterization of the relationship between continuous noise, one-shot noise, learning rates, and the absence of corrective saccades.

It can also be noted that the speed with which the model converges to stability is a function of the learning rate, 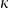. Increasing this learning rate may help overcome larger amounts of noise, but there is also the potential for introducing learning instabilities with larger learning rates. The model as presented is tuned approximately at the same speed as the goldfish (see, e.g., figure 8). The empirical consequences of varying learning rate could be predicted, if methods for manipulating such rates in the biological system could be established.

A related empirical question that arises given this model is: How are corrective and intentional saccades distinguished? In the model, that distinction is made by filtering based on the magnitude of the velocity command. However, it remains an open question what the biological mechanism underlying this filtering might be. This issue is left largely unaddressed here because there are several potential means of identifying corrective saccades. For example, the learning process may require a kind of “activation energy” to initiate learning, in which case large saccades would reduce this energy and act as inhibitors for learning. It is also possible that the (amplitude independent) frequency content of saccades is used to trigger the learning process, such that intentional saccades do not cause modification of the synaptic weights. As well, the duration of the saccades can be used as a means of distinguishing intentional from corrective saccades. In the end, the magnitude filtering implemented in this study was chosen because of simplicity and lack of experimental evidence for any one of these potential mechanisms.

Notably, our particular choice of filtering method does not seem crucial. We have run single simulations with other filtering methods with similar results. For example, using the filtering by change in position (see Figure 2), the time constant of the network improved from 5.7 s to 69.5 s, similar to our chosen method. More importantly, however, the learning rule itself is independent of the method of distinguishing corrective from intentional saccades, although it assumes there is some mechanism that provides this distinction.

### The generalized learning rule

Consideration of the generalized learning rule raises interesting possibilities that could be tested experimentally. Perhaps most speculatively, the rule suggests that intrinsic neuron properties play a central role in a how a particular neuron is exploited by a system. The encoding vector 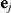 and gain 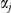 determine how the error signal is “interpreted” by a given neuron. The mapping of input currents onto neural activity are a function of intrinsic neuron properties, like the membrane resistance and capacitance, channel density, dendritic morphology and so on. This suggests it may be possible to experimentally determine relationships between such properties and how cells are exploited in a given learning circuit.

Much less speculatively, the general structure of the rule suggests simple behavioral experiments. For example, if an error signal analogous to retinal slip is available to head-direction, path integration systems, or working memory systems, it should be possible to similarly mis-tune those systems with careful manipulation of the stimulus. If such mis-tuning is achievable, it would suggest that this kind of plasticity is broadly important for the neural control of behavior.

Returning to the saccadic system specifically, it is evident that the error signal is generated by elements of the oculomotor system external to the integrator itself. However, it is clearly the case that such a signal is self-generated by the neurobiological system as a whole (as captured by the OMS model). This signal allows for a kind of “self-directed organization” of the system. The generalization suggests that any other error signal that can be self-generated can also be exploited by this rule for tuning a network to perform other kinds of computations. Preliminary results show that this generalized rule is able to learn arbitrary non-linear vector transformations [62]. Notably, the generalization of the error signal and the neural representation does not change the basic gated pseudo-Hebbian nature of the rule.

In addition, clear differences in the consequences of different kinds of learning arise in the case of stability. A supervised rule, such as backpropagation through time [63], requires enforcing the desired output on the state of the system (i.e., eye position), which is biologically implausible in this case as the correcty eye position is not immediately available to the integrator. An unsupervised stability rule [32] will enforce stability over the range of experienced input. Thus the dynamics of the system (i.e., whether it is stable, unstable, or damped) are determined by the rule itself. In contrast, a self-directed rule, like that presented here, determines the stability of the system in an environmentally dependent way. As demonstrated by the mis-tuning experiments, the stability of the integrator is not intrinsic, but rather tied to environmental stability. Or, more accurately, tied to the system's ability to generate corrective stability signals based on environmental cues. Uncertainties regarding environmental dynamics may make it evolutionarily advantageous to prefer rules that rely on self-directed organization in many circumstances.
